# Coresidence increases the risk of testing positive for COVID-19 among older Brazilians

**DOI:** 10.1186/s12877-022-02800-6

**Published:** 2022-02-05

**Authors:** Flavia Cristina Drumond Andrade, Nekehia T. Quashie, Luisa Farah Schwartzman

**Affiliations:** 1 School of Social Work, University of Illinois at Urbana-Champaign, 1010 W. Nevada, Urbana, IL 61801 USA; 2 Department of Health Studies, University of Rhode Island Independence Square, 25 West Independence Way, Kingston, RI 02881 USA; 3 Department of Sociology, University of Toronto, 3359 Mississauga Road, Mississauga, ON L5L 1C6 Canada

**Keywords:** Living arrangements, Older adults, Brazil, COVID-19 symptoms

## Abstract

**Background:**

Brazil is among the countries hit hardest by COVID-19, and older adults are among the vulnerable groups. Intergenerational coresidence and interdependence among family members, both prevalent in Brazil, likely increase social and physical contact and thus potential infection.

**Methods:**

Using nationally representative data from the COVID-19 module of the Brazilian National Household Sample Survey (Pesquisa Nacional por Amostra de Domicílios), collected between July and November of 2020, we examined the association between living arrangements and exposure to and testing for COVID-19 among 63,816 Brazilians aged 60 years and older. We examine whether living arrangements influence self-reported COVID-19 symptoms as an indicator of subjective health assessment, testing as an indicator of health care service use, and a positive COVID-19 test result as an objective indicator of exposure to the disease.

**Results:**

Living arrangements shape older adults’ vulnerabilities to COVID-19 exposure and testing. Specifically, those living alone were more likely to report having symptoms and having had a test for COVID-19. However, older adults in multigenerational and skipped generation households were more likely than solo-dwellers to test positive for COVID-19. Those with symptoms were more likely to test, regardless of their living arrangement. Among older adults without symptoms, those living alone had a higher probability of testing than those living in multigenerational or skipped-generation households.

**Conclusions:**

Overall, our findings suggest that coresidence with younger family members puts older adults’ health at risk in the context of COVID-19. As younger Brazilians are increasingly vulnerable to COVID-19 and experiencing severe outcomes, policy makers need to be more attentive to the health needs of households that comprise older and younger cohorts, which are also more prevalent in poor and marginalized segments of the population.

## Background

The Brazilian federal government has adopted a denialist policy toward COVID-19 and lacked a coordinated national strategy to slow the spread of the virus. While subnational governing bodies enacted mitigation measures to reduce social contact through physical distancing to slow transmission, these efforts have often been inconsistent or ineffective [1]. As a result, many Brazilians, even those with COVID-19 symptoms, have misunderstood, ignored, or been unable to follow social distancing guidance or adjust their behavior to limit the spread of the virus in any other way [1]. This has had severe consequences: Brazil is among the countries hit hardest by COVID-19. By the end of June 2021, the country had over 18.4 million cases, 513,000 deaths, and a high incidence of very contagious variants [2]. Older adults, defined as people ages 60 and older [3], were among the hardest hit [4], representing over 70% of COVID-19 deaths [5].

Coresidence with family is one of the most salient forms of family support [6]. Intergenerational coresidence often reflects the needs and resources of older and younger generations and facilitates ease of support exchanges between family members. Younger generations provide care for older adults with chronic conditions or limitations with activities of daily living [7, 8], while older adults may contribute money, housing, and childcare [9, 10]. Estimates from the 2010 Brazilian Census indicate that approximately 51% of Brazilians 60 years and older live with their adult children, while 22% live with their spouse only and 13.3% live alone [11].

It is well established that social relationships, as a source of social connection and support, are important for individuals’ health and health behaviors across the life course [12, 13]. Prior research in Latin American and Caribbean countries shows that support exchanges with children and extended family can benefit older adults’ health [14, 15]. However, the pandemic may have outweighed these benefits by raising the risk of COVID-19 exposure among those who coreside with family [16]. On the other hand, those who lived alone may have increased risks of poor mental and physical health due to a lack of support and social isolation [17]. Nevertheless, research conducted during the early stages of the pandemic in Brazil showed that adults aged 60 or older were more likely than adults ages 18 to 59 to adhere to preventive measures [18] and to stay home [19].

To our knowledge, no study has specifically focused on the role of the household context for older Brazilians’ exposure to COVID-19. Given the combination of the ongoing public health crisis and the importance of social relationships for well-being in Brazil, this study addresses the following pertinent question: Which living arrangements present the most health risks for older Brazilians in the context of the COVID-19 pandemic? We use nationally representative data from the COVID-19 module of the Brazilian National Household Sample Survey (Pesquisa Nacional por Amostra de Domicílios, PNAD), collected from July to November 2020 to address this question. We examined whether older adults’ living arrangements influence their self-reported COVID-19 symptoms as an indicator of subjective health assessment, testing as an indicator of COVID-19 health care service use, and positive results of COVID-19 as an objective indicator of exposure to the disease. Results indicated that different household contexts could be a health resource or risk for older Brazilians during the ongoing pandemic.

### Living arrangements and older adults’ health

Coresidence with adult children and other family members is the prevailing living arrangement for older adults in many developing countries [20], including Brazil [21]. Limited state-provided safety nets for older adults, rendering family the main facilitator of financial access to care, may make coresidence with family members the ideal living arrangement to meet older adults’ needs and sustain their well-being [22, 23]. In this study, we conceive living arrangements as a form of structural support representing the potential for social interaction and access to support to maintain health.

Living with children and other family members facilitates the exchange of various forms of social support, including interpersonal contact as well as financial, emotional (e.g., sharing concerns), and instrumental support, which reduce older adults’ risks of social isolation and poor health [24–26]. Family members can also be a source of social control to regulate health behaviors or influence that inspires a sense of responsibility for one’s health [27, 28] that may be reinforced by coresidence. On the other hand, coresidence can be a source of distress if family relationships are strained or unsupportive [29], undermining older adults’ health. Thus, even outside the issue of infectious disease, coresidence with family members may not be universally beneficial.

Indeed, empirical studies on the health benefits (physical and mental health) of coresidence for older adults have yielded mixed results. In developing countries within Asia, Latin America, the Caribbean, and sub-Saharan Africa, where multigenerational living arrangements are normative, older adults living alone tend to be more vulnerable to poor health, including depression and short-term illness, relative to those in multigenerational households [30–32]. At the same time, research on Chinese older adults found multigenerational living does not lower risk of depression among older adults [30]. Similarly, skipped-generation households, where older adults live with grandchildren only and are the primary caregivers, can present health risks and benefits. Research among older adults in China, where grandparent caregiving is socially expected, suggests that grandparents in skipped generation households experience slower health declines than grandparents with other living arrangements [33]. However, studies among US older adults suggest that grandparents who are primary caregivers or provide intensive grandchild care are more vulnerable to poor health, including respiratory infections [34], as well as declines in mental health and healthy habits [35].

The empirical evidence regarding the importance of coresidence as a source of family support to meet older adults’ health care needs in developing societies is also inconclusive. This, in part, reflects differences in country context and the dimension of health examined (e.g., health care utilization, health expenditure). Li and Chi (2011), in their analysis of Chinese older adults’ health service utilization, found that intergenerational coresidence was associated with a lower likelihood of doctors’ visits, which may reflect a higher level of home-based support by children that substitutes for formal care [36]. However, coresidence among older adults in India was associated with a higher probability of assistance with medical expenses and providing support when hospitalized [37]. Research on coresidence and its association with health in Brazil is sparse. Faustino and colleagues (2020) examined differences in household incomes after Brazil’s social security expansion and found that households with older adults tend to have higher incomes and higher personal health expenditure than households without older adults [38].

Beyond the uncertainty created by mixed results prior to the pandemic, the COVID-19 pandemic presented a novel situation that raises questions about how exposure and morbidity among older adults may vary across living arrangements. This is a vital question given the enormous toll on Brazil’s older adult population, the unresolved nature of the pandemic as of this writing, and the threat of future global pandemics.

### Living arrangements and older adults’ health during the pandemic

Research in the United States suggests that the context in which older adults live has implications for their behaviors, adherence to guidelines, exposure, complications, and death due to COVID-19 [39]. Given that COVID-19 mitigation measures in Brazil revolved around minimizing social contacts, especially with non-household members, older adults’ living arrangements are a critical context to examine their risks of exposure and health behaviors related to the virus.

As most Brazilian older adults receive care from family members, solo-dwelling older adults in Brazil may have become more aware of their lack of close social ties and need to be more self-reliant. For instance, older adults who live independently usually report higher levels of loneliness [40]. Although loneliness seems to have decreased in the early stages of the pandemic (data from May–June 2020) in Brazil compared to pre-pandemic levels, there is evidence it increased in the ensuing months as the pandemic continued [41]. Solo dwellers may have left their homes as a necessary measure to maintain social engagement [42]. However, social distancing guidelines likely made it difficult to interact and access goods and services for daily living. Research conducted during the early stages of the pandemic found that among older Brazilians who needed assistance with daily activities, those who received such assistance from coresident and non-resident caretakers were more likely to remain socially distanced [7]. Given that coresidence is typically the easiest way to access such support, older adults living in intergenerational households may have been better able to adhere to social distancing guidelines than those living alone.

Indeed, early empirical evidence on older adults’ COVID-19 exposure and health behaviors as linked to their living arrangements suggests that in countries with strong social norms of intergenerational contacts, such as Italy, COVID-19 was more prevalent in areas with a higher prevalence of solo-dwelling older adults [43]. Similarly, older adults in Israel who lived alone were less likely to adopt protective measures, such as using face masks and avoiding social interactions with family and friends, than those living intergenerationally [42]. In Mexico, solo-dwelling has been identified as a risk factor for COVID-19 positivity among older adults [44].

Living arrangements may also predict vulnerability to morbidity and mortality from COVID-19. Older adults who live alone in Brazil are more likely to have worse health conditions and lower socioeconomic status than older adults who live with others [45], both of which are risk factors for hospitalization and death from COVID-19. Poorer and less educated older Brazilians have worse health outcomes, including chronic conditions and frailty, than those with better socioeconomic conditions [46, 47]. These vulnerabilities intersect with the weathering process, in which individuals with disadvantaged socioeconomic conditions face higher health risks that accumulate over the life course [48]. However, the impact of these risks is uncertain; solo-dwelling older adults with worse health may have increased adherence to social distancing guidelines because they understand the risks. Alternatively, lower socioeconomic status may increase the need to venture out to meet daily needs.

Conversely, older adults living in multigenerational living arrangements, many times in crowded environments, are more likely to have heightened exposure and transmission rates of COVID-19 [16, 39]. A study of COVID-19 mortality conducted during the early stages of the pandemic among older adults in Sweden showed older adults who lived with working-age adults were at higher risk of COVID-19 mortality than older adults residing with older adults only [49]. In Brazil, as many schools closed in the spring of 2020, older adults in multigenerational households may have had heightened risks of exposure to the virus due to extended time with children and adolescents at home [50]. Additionally, pandemic precautions may have increased sedentary behaviors at home, potentially increasing chronic conditions that raise the risk of severe COVID-19 [51].

Overall, the theoretical and empirical literature suggests that the relationship between living arrangements and exposure to COVID-19 is not straightforward for older adults. Solo dwelling and coresidence with younger relatives may both increase older adults’ exposure to COVID-19. Our study investigates the overall direction of the relationship between living arrangements of older adults (i.e., living alone, with older adults only, multigenerational, skipped generation, and with adults and older adults, but not children), and vulnerability to COVID-19 empirically, as measured by self-reported symptoms and positive test results. Given that exposure assessments often depend on testing, we further investigate testing rates to indicate whether coresidence with family members may encourage health-seeking as indicated by COVID-19 testing. Furthermore, as symptoms may drive testing and, consequently, positive results, we examine whether reporting symptoms modify the association between living arrangements on COVID-19 testing and having a positive result.

## Methods

### Data

Individual-level data from the COVID-19 Pesquisa Nacional por Amostra de Domicílios (PNAD) were used in the analyses. The COVID-19 PNAD is a telephone surveillance survey module within the Continuous National Household Survey (PNAD-C), which focuses on the impact of the pandemic on the labor market [52]. It was conducted by the Brazilian Institute of Geography and Statistics (Instituto Brasileiro de Geografia e Estatística, IBGE) in partnership with the Ministry of Health to monitor COVID-19 related health indicators across time and geographic areas through monthly surveys. IBGE performed data collection in accordance with the relevant national guidelines and regulations. All participants consented to participate in the COVID-19 PNAD. The module was designed to assess the impact of COVID-19 on work and health indicators at the national, geographic macro-region, and state levels [52].

In order to obtain the sample for the COVID-19 PNAD, IBGE used the base of 211,000 households that participated in the PNAD-C in the first quarter of 2019 and selected 193,662 households with a registered telephone number. This represents 92% of the basic sample. The sample is fixed, and selected households interviewed in the first wave remained in subsequent months until the last wave of data collection [53]. The first wave of data collection was in May of 2020, and the last one was in November of 2020.

The resident who answered the phone provided information on behalf of all other residents in a household [52]. Questions focused on flu-like symptoms in the previous week, access to health care and health care utilization, and the household’s socioeconomic conditions [52]. Questions on testing and positivity were introduced in July 2020. Therefore, we used the July–November data. Further details regarding the COVID-19 PNAD, survey design, and questionnaires are available on the Brazilian Census Bureau website (www.ibge.gov.br).

Our study makes use of the longitudinal feature of the COVID-19 PNAD. Given the IBGE did not provide identifiers to link individuals’ records over time [54], we use demographic variables and information in the household to link residents’ records over time. This involved merging files using the command “reclink2” [55], which performs probabilistic linkage of the records. We use the variables that identify the households based on the primary sampling unit and the household selection number, and individuals’ sex and date of birth to link the records over time. The July 2020 survey interviewed 384,166 individuals, of which 66,253 were older adults. Using a modified version of the protocol developed by Teixeira-Junior and colleagues [54], we longitudinally link 83.27% of the participants, yielding a sample of 319,906. We next eliminated those who were younger than 60 years (63,945 older adults). We also restricted the analyses to those with complete data on all selected variables. The final analytic sample consists of 63,816 adults (60 years and older), providing 295,696 observations across all months, which we consider adequate for our research questions. We use STATA SE 16.1 for all analyses.

Oral consent was obtained for all respondents. Data are publicly available on the Internet, with all identifying factors removed. All methods used in this study are based on this anonymized secondary data from the COVID-19 PNAD. As per the Office for Protection of Research Subjects at the University of Illinois at Urbana-Champaign, the current study is considered exempt research*.*

### Measures

#### COVID-19 exposure

The primary outcome variables were whether the older adult reported having a COVID-19 sign or symptom, being tested, and/or testing positive between July and November 2020.

##### COVID-19 symptoms

The 12 symptoms mentioned in the survey were fever, cough, sore throat, difficulty breathing, headache, chest pain, nausea, stuffy or runny nose, fatigue, eye pain, loss of smell or taste, and muscle pain. We created a dichotomous variable to indicate whether the participant reported any of these symptoms in the preceding week.

##### Testing

Each month, information on testing was collected using the following question “Did you take any tests to find out if you were infected with the coronavirus?” Follow-up questions inquired about the nature of the tests: “Was the test collected with a swab in the mouth and/or nose swab?” “Did you take the blood collection test through a finger stick?” and “Did you take the blood collection test through an arm vein?” We created a dichotomous variable to indicate whether the participant had any test or not.

##### Positive result

After each question related to the nature of the test, participants were asked about the result: “What was the result?” Participants who indicated they received a positive result on any of these tests were classified as having a positive test. Participants who received a negative test include those who either indicated they had received a negative test for all tests, were still awaiting their results, or they had received an inconclusive result. We created a dichotomous variable to examine whether the participant had received a positive test or not, for any one of the methods of COVID-19 tests.

#### Living arrangements

The primary independent variable is living arrangements. To construct this variable, we used the age of all the residents in the household. For this study, we defined older adults as persons aged 60 and older, adults ages 18–59, and children ages 17 and younger. In Brazil, the age of majority (or threshold of adulthood) as recognized in law is 18. Living arrangements were categorized as 1) older adults living alone in the household, 2) households with older adults only, 3) multigenerational households including children, adults, and older adults, 4) skipped generation households including children and older adults but not adults, and 5) households including adults and older adults, but not children.

#### Covariates

Brazil has high social inequalities in living conditions, both across individuals and geographic regions [56], which the pandemic has exacerbated. COVID-19 mortality rates have been higher in Brazil’s North and Northeast regions [4, 57], which are less economically developed than Southern areas [58]. Income inequalities in health care access are also evident [59]. Thus, we accounted for household income measures as per capita household income quartiles, geographic region (North, Northeast, Center-West, Southeast, and South), and rural/urban residence. Furthermore, the pandemic disrupted access to health care services. Primary health care units were reorganized to prioritize respiratory problems and some individuals suspended care related to diabetes and hypertension management, both of which increased risk from COVID-19 [60]. Older Brazilians with chronic conditions were more likely than those with no health conditions to cancel medical appointments and care during the pandemic [61]. Thus, we also accounted for medical conditions (diabetes, hypertension, heart disease, asthma/bronchitis/emphysema, cancer, and depression), which increase mortality [62].

We also included individuals’ sociodemographic characteristics, age in years, gender (male, female), education (without instruction, less than high school, complete high school, and college education or above), and whether the individual responded to the survey or used a proxy respondent, and race/color of the participant. IBGE classifies Brazilian racial/color categories into *preto*, *pardo, branco, amarelo,* and *indígena*. While *preto* and *branco* can be literally translated into Black and White, and *pardo* (which we translate here as Brown) is a category generally understood as applying to people who are considered mixed-race between Black and White. Besides considerations of ancestry and appearance, other factors such as political orientation, social class, and geographic location may also influence classification [63, 64]. We translate the category *indigena* as Indigenous. The category *amarela* (literally “yellow,” or Asian) is included together with “other” due to its small size.

### Statistical analysis

**Fig. 1:**
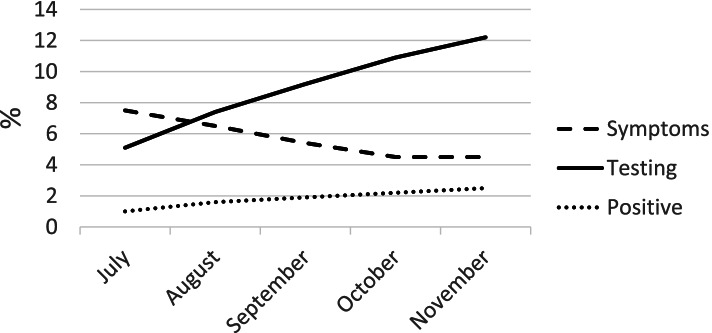
Trends in reported COVID-19 symptoms, testing, and positive cases among older adults in Brazil, July–November 2020

We first produced descriptive statistics for characteristics of the sample utilizing derived sampling weights to account for the sample design (Table 1). Figure 1 presents the prevalence of symptoms, testing, and positive results among older adults during the survey months.

**Table 1:** Characteristics of participants aged 60 and older of the COVID-19 PNAD, by living arrangements, July 2020

	Total	Older adults alone (14.1%)	Older adults only (29.1%)	Multigenerational (14.5%)	Skipped generation (2.2%)	Adults and older adults only (40%)	*p*-value
Mean household size	2.7	1.0	2.1	4.8	2.9	3.0	< 0.0001
Mean age	70.1	71.6	70.6	69.1	67.9	69.6	< 0.0001
Female (%)	55.9	68.9	52.1	55.3	62.1	54.0	< 0.0001
Race (%)							< 0.0001
White	51.6	55.0	59.6	39.7	35.2	49.9	
Black	8.2	8.0	6.1	11.3	8.8	8.5	
Brown	38.7	35.7	32.7	47.7	54.6	40.0	
Indigenous	0.3	0.3	0.2	0.4	0.3	0.2	
Other	1.2	1.0	1.4	0.8	1.0	1.3	
Education (%)							< 0.0001
Without schooling	13.1	11.9	10.3	18.7	17.8	13.2	
Less than HS	56.1	54.6	56.0	58.4	64.8	55.4	
HS complete	18.3	18.5	18.7	15.8	12.0	19.1	
College or more	12.6	15.0	15.0	7.1	5.4	12.3	
Household per capita income quartile (%)							< 0.0001
1	12.7	2.9	6.4	35.6	38.1	11.0	
2	19.4	3.3	11.6	33.6	36.1	24.7	
3	39.0	49.2	50.1	20.3	18.6	35.3	
4	28.9	44.6	32.0	10.5	7.2	29.0	
Region (%)							< 0.0001
North	5.3	3.7	3.6	10.5	10.0	5.1	
Northeast	24.1	19.9	19.2	31.8	36.1	25.6	
Southeast	47.4	51.9	50.2	38.6	35.9	47.6	
South	16.7	18.3	21.0	11.8	10.4	15.0	
Center West	6.5	6.2	5.9	7.3	7.8	6.7	
Urban (%)	86.4	89.7	84.5	85.0	80.2	87.4	< 0.0001
Health conditions (%)							
Diabetes	20.9	21.4	21.1	20.5	24.3	20.4	0.0461
Hypertension	46.1	50.0	45.3	45.9	47.2	45.3	< 0.0001
Heart disease	11.0	12.8	11.5	9.5	9.3	10.6	< 0.0001
Asthma	6.0	8.3	5.7	5.5	6.0	5.6	< 0.0001
Cancer	3.9	4.9	4.3	2.9	2.5	3.7	< 0.0001
Depression	5.5	9.2	5.3	4.3	4.5	4.9	< 0.0001
Proxy respondents (%)	57.7	20.4	56.5	74.3	48.9	66.2	< 0.0001

Source: Authors’ calculations using data from the COVID-19 PNAD, July. Weighted estimates

**Fig. 2:**
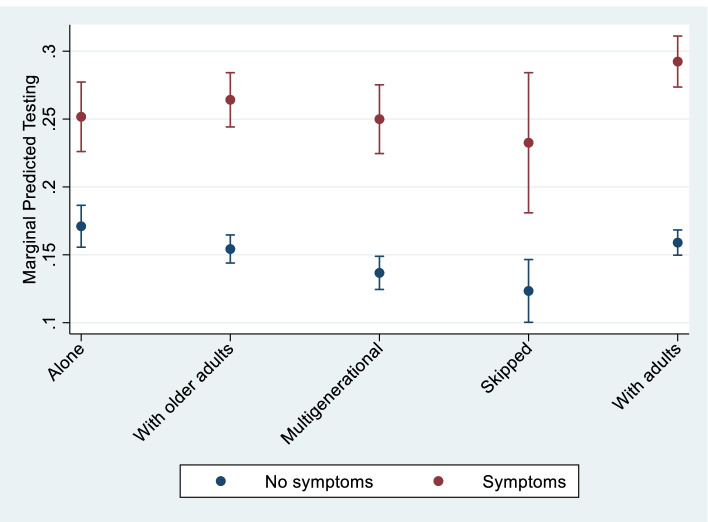
Predicted probabilities of testing among older adults by living arrangements and reporting of symptoms in Brazil, July–November 2020

Mixed-effects Poisson regression was used to examine the association between living arrangements and the COVID-19 outcome measures. We included random effects to account for within-cluster homogeneity in our outcomes and estimate robust standard errors. We provided estimates of the adjusted prevalence rate ratios to assess the influence of living arrangements on COVID-19 outcomes for the entire sample (Table 2). We further examined whether reporting symptoms moderated the association between living arrangements and each COVID-19 related test—overall testing and receiving positive COVID-19 results (Table 3). To facilitate the interpretation of regression results in Table 3, particularly the interaction effects, we measured the contrasts involving factor variables and their interactions using the “contrast” command and the linear predictions obtained with the “margins” command. We used the “marginsplot” command to graph the effect of the symptoms and living arrangements on testing. Results appear in Fig. 2.

**Table 2:** Adjusted Prevalence Ratios and 95% confidence intervals (CI) for factors associated with the reporting of COVID-19 symptoms, testing and receiving a positive result, older adults, Brazil (July–November)

	Symptoms		Testing		Positive	
	PR	95% CI	PR	95% CI	PR	95% CI
*Fixed*						
Living arrangement (versus older adult alone)						
Older adults only	0.726***	(0.68, 0.77)	0.910	(0.82, 1.01)	1.013	(0.80, 1.29)
Multigenerational	0.722***	(0.67, 0.78)	0.815***	(0.73, 0.92)	1.532**	(1.15, 2.04)
Skipped generation	0.775***	(0.68, 0.88)	0.729**	(0.60, 0.89)	1.607*	(1.04, 2.48)
Adults and older adults	0.735***	(0.69, 0.78)	0.945	(0.86, 1.04)	1.269*	(1.00, 1.61)
Age (in years)	1.000	(1.00, 1.00)	0.984***	(0.98, 0.99)	0.975***	(0.97, 0.98)
Female (versus male)	1.019	(0.98, 1.06)	0.913**	(0.86, 0.98)	0.882	(0.75, 1.03)
Race (versus White)						
Black	1.088*	(1.01, 1.17)	0.945	(0.84, 1.07)	0.929	(0.70, 1.24)
Brown	1.091***	(1.04, 1.14)	0.925*	(0.86, 0.99)	1.138	(0.96, 1.35)
Indigenous	1.493**	(1.13, 1.97)	1.813**	(1.21, 2.71)	1.895	(0.48, 7.55)
Other	1.011	(0.81, 1.26)	0.921	(0.68, 1.25)	0.496	(0.22, 1.12)
Education (versus no schooling)						
Less than high school	0.969	(0.91, 1.03)	1.082	(0.98, 1.20)	1.200	(0.97, 1.49)
High school complete	0.892**	(0.83, 0.96)	1.579***	(1.40, 1.78)	1.942***	(1.47, 2.56)
College or more	0.839***	(0.77, 0.92)	2.561***	(2.26, 2.90)	2.594***	(1.86, 3.61)
Per capita household quartile (versus 1st)						
2	0.972	(0.92, 1.02)	1.029	(0.97, 1.09)	1.066	(0.94, 1.20)
3	0.953	(0.90, 1.01)	1.174***	(1.10, 1.25)	1.255**	(1.09, 1.45)
4	0.895***	(0.84, 0.96)	1.464***	(1.36, 1.57)	1.541***	(1.31, 1.82)
Region (versus North)						
Northeast	0.893**	(0.83, 0.96)	0.819***	(0.73, 0.92)	0.345***	(0.24, 0.50)
Southeast	0.781***	(0.73, 0.84)	0.435***	(0.39, 0.49)	0.112***	(0.08, 0.16)
South	0.916*	(0.84, 0.99)	0.345***	(0.30, 0.39)	0.0778***	(0.05, 0.11)
Center West	0.972	(0.89, 1.06)	0.948	(0.84, 1.07)	0.466***	(0.30, 0.72)
Urban (versus rural)	1.016	(0.97, 1.07)	2.492***	(2.24, 2.77)	2.404***	(1.98, 2.92)
Health conditions (versus none)						
Diabetes	1.236***	(1.18, 1.29)	1.227***	(1.15, 1.31)	1.538***	(1.33, 1.78)
Hypertension	1.267***	(1.22, 1.32)	1.115***	(1.06, 1.17)	1.324***	(1.18, 1.48)
Heart disease	1.494***	(1.42, 1.57)	1.330***	(1.24, 1.42)	1.253**	(1.06, 1.48)
Asthma	1.998***	(1.89, 2.12)	1.552***	(1.44, 1.68)	1.495***	(1.22, 1.83)
Cancer	1.364***	(1.25, 1.48)	1.228***	(1.10, 1.36)	1.284	(0.98, 1.69)
Depression	1.771***	(1.67, 1.88)	1.237***	(1.14, 1.34)	1.468***	(1.20, 1.80)
Proxy respondent (versus no)	0.608***	(0.58, 0.63)	0.934***	(0.90, 0.97)	1.024	(0.94, 1.12)
Month (versus July)						
August	0.888***	(0.86, 0.92)	1.389***	(1.35, 1.43)	1.513***	(1.41, 1.62)
September	0.735***	(0.71, 0.77)	1.739***	(1.69, 1.79)	1.949***	(1.82, 2.09)
October	0.735***	(0.57, 0.63)	2.068***	(2.00, 2.14)	2.266***	(2.11, 2.43)
November	0.599***	(0.58, 0.63)	2.331***	(2.26, 2.41)	2.496***	(2.32, 2.68)
Constant	0.605***	(0.05, 0.07)	0.007***	(0.00, 0.01)	0.000***	(0.00, 0.00)
Random ID	1.147	(1.10, 1.20)	6.783	(6.65, 6.92)	11.247	(10.83, 11.68)

Source: Authors’ calculations using data from the COVID-19 PNAD, July–November. Robust standard errors have been estimated and account for the clustering of data on individuals

Note: * *p* < 0.05, ** *p* < 0.01, *** *p* < 0.001, 95% CI: 95% confidence interval

**Table 3:** Adjusted prevalence ratios and 95% confidence intervals (CI) for factors associated with the reporting of COVID-19 testing and receiving a positive result, Brazil

	Testing (M1)		Testing (M2)		Positive (M1)		Positive (M2)	
	PR	95% CI	PR	95% CI	PR	95% CI	PR	95% CI
*Fixed*								
Living arrangement (versus older adult alone)								
Older adults only	0.921	(0.83, 1.02)	0.902*	(0.81, 1.00)	1.021	(0.81, 1.29)	1.012	(0.80, 1.29)
Multigenerational	0.821***	(0.73, 0.92)	0.799***	(0.71, 0.90)	1.522**	(1.16, 2.00)	1.506**	(1.14, 2.00)
Skipped generation	0.743**	(0.61, 0.90)	0.721**	(0.59, 0.88)	1.634*	(1.06, 2.51)	1.587*	(1.02, 2.48)
Adults and older adults	0.955	(0.87, 1.05)	0.930	(0.84, 1.02)	1.292*	(1.03, 1.63)	1.265	(1.00, 1.60)
Symptoms	1.734***	(1.68, 1.79)	1.472***	(1.37, 1.58)	2.357***	(2.21, 2.52)	2.133***	(1.82, 2.50)
Interactions								
Symptoms*older adults only			1.164**	(1.06, 1.28)			1.064	(0.87, 1.30)
Symptoms*Multigenerational			1.243***	(1.12, 1.38)			1.078	(0.87, 1.33)
Symptoms*Skipped generation			1.281*	(1.05, 1.56)			1.184	(0.89, 1.58)
Symptoms*Adults and older adults			1.250***	(1.15, 1.36)			1.170	(0.98, 1.40)
Age (in years)	0.984***	(0.98, 0.99)	0.984***	(0.98, 0.99)	0.976***	(0.97, 0.99)	0.976***	(0.97, 0.99)
Female (versus male)	0.911**	(0.85, 0.97)	0.911**	(0.85, 0.97)	0.882	(0.76, 1.03)	0.882	(0.76, 1.03)
Race (versus White)								
Black	0.946	(0.84, 1.07)	0.946	(0.84, 1.07)	0.931	(0.71, 1.23)	0.931	(0.71, 1.23)
Brown	0.925*	(0.86, 0.99)	0.925*	(0.86, 0.99)	1.129	(0.96, 1.33)	1.129	(0.96, 1.33)
Indigenous	1.780**	(1.19, 2.67)	1.778**	(1.18, 2.67)	1.783	(0.49, 6.48)	1.784	(0.49, 6.48)
Other	0.938	(0.69, 1.27)	0.938	(0.69, 1.27)	0.503	(0.23, 1.11)	0.503	(0.23, 1.11)
Education (versus no schooling)								
Less than high school	1.081	(0.98, 1.20)	1.080	(0.97, 1.20)	1.201	(0.97, 1.48)	1.200	(0.97, 1.48)
High school complete	1.576***	(1.40, 1.78)	1.574***	(1.40, 1.77)	1.948***	(1.49, 2.54)	1.945***	(1.49, 2.54)
College or more	2.558***	(2.26, 2.89)	2.556***	(2.26, 2.89)	2.536***	(1.85, 3.47)	2.534***	(1.85, 3.47)
Per capita household quartile (versus 1st)								
2	1.033	(0.97, 1.09)	1.033	(0.97, 1.09)	1.088	(0.96, 1.23)	1.087	(0.96, 1.23)
3	1.179***	(1.10, 1.26)	1.178***	(1.10, 1.26)	1.264**	(1.09, 1.46)	1.264**	(1.09, 1.46)
4	1.469***	(1.37, 1.58)	1.469***	(1.37, 1.58)	1.558***	(1.32, 1.83)	1.554***	(1.32, 1.83)
Region (versus North)								
Northeast	0.825***	(0.74, 0.92)	0.825***	(0.74, 0.92)	0.369***	(0.26, 0.51)	0.369***	(0.26, 0.51)
Southeast	0.442***	(0.39, 0.49)	0.442***	(0.39, 0.49)	0.123***	(0.09, 0.17)	0.123***	(0.09, 0.17)
South	0.348***	(0.31, 0.40)	0.349***	(0.31, 0.40)	0.0846***	(0.06, 0.12)	0.0847***	(0.06, 0.12)
Center West	0.948	(0.84, 1.07)	0.948	(0.84, 1.07)	0.479***	(0.32, 0.71)	0.479***	(0.32, 0.71)
Urban (versus rural)	2.458***	(2.22, 2.73)	2.457***	(2.22, 2.72)	2.333***	(1.93, 2.82)	2.332***	(1.93, 2.82)
Health conditions (versus none)								
Diabetes	1.214***	(1.14, 1.29)	1.213***	(1.14, 1.29)	1.478***	(1.28, 1.71)	1.479***	(1.28, 1.71)
Hypertension	1.106***	(1.05, 1.16)	1.106***	(1.05, 1.16)	1.319***	(1.18, 1.47)	1.320***	(1.18, 1.47)
Heart disease	1.310***	(1.22, 1.40)	1.310***	(1.23, 1.40)	1.229*	(1.04, 1.45)	1.231*	(1.04, 1.45)
Asthma	1.503***	(1.39, 1.62)	1.505***	(1.39, 1.63)	1.479***	(1.21, 1.81)	1.484***	(1.22, 1.81)
Cancer	1.219***	(1.10, 1.36)	1.221***	(1.10, 1.36)	1.298	(0.99, 1.70)	1.302	(0.99, 1.71)
Depression	1.211***	(1.11, 1.32)	1.211***	(1.11, 1.32)	1.404**	(1.15, 1.72)	1.404**	(1.15, 1.72)
Proxy respondent (versus no)	0.945**	(0.91, 0.98)	0.946**	(0.91, 0.98)	1.042	(0.95, 1.14)	1.040	(0.95, 1.14)
Month (versus July)								
August	1.408***	(1.37, 1.45)	1.409***	(1.37, 1.45)	1.584***	(1.48, 1.69)	1.585***	(1.48, 1.69)
September	1.795***	(1.74, 1.85)	1.797***	(1.74, 1.85)	2.121***	(1.98, 2.27)	2.124***	(1.98, 2.28)
October	2.167***	(2.10, 2.24)	2.169***	(2.10, 2.24)	2.579***	(2.40, 2.77)	2.581***	(2.40, 2.77)
November	2.450***	(2.37, 2.53)	2.453***	(2.37, 2.53)	2.909***	(2.70, 3.13)	2.912***	(2.70, 3.14)
Constant	0.001***	(0.00, 0.01)	0.007***	(0.00, 0.01)	0.000***	(0.00, 0.00)	0.001***	(0.00, 0.00)
Random ID	6.577	(6.44, 6.72)	6.571	(6.43, 6.71)	10.651	(10.25, 11.07)	10.648	(10.25, 11.07)

Source: Authors’ calculations using data from the COVID-19 PNAD, July–November. Robust standard errors have been estimated and account for the clustering of data on individuals

Note: * *p* < 0.05, ** *p* < 0.01, *** *p* < 0.001, 95% CI: 95% confidence interval

Last, we conducted sensitivity analyses using cumulative measures for the dependent variables. To do so, for each measure, we combined affirmative response in any of the waves and created a cumulative measure across the five waves for symptoms, testing, and positive results. In these additional analyses, we used logistic regression to examine the association between living arrangements and COVID-19 outcomes. Covariates and living arrangements values from July were used in these analyses.

## Results

### Descriptive analysis

Figure 1 shows the prevalence of COVID-19 symptoms, testing, and positive tests over July to November 2020. The prevalence of symptoms among older adults decreased between July and November 2020, which aligns with the decline in the overall number of cases in the country. Testing increased from July to November 2020. While this is an important measure to safeguard the health of older adults and their families, it may also have influenced the increasing prevalence of positive reports over time as cases were identified.

Table 1 shows descriptive statistics for individuals’ sociodemographic characteristics, geographic region, and health for the entire sample, stratified by living arrangements. There is substantial variation in socioeconomic and health conditions across living arrangements. Women are more likely to live alone or to live with their grandchildren. White older adults are more likely to live independently or with other older adults, whereas Brown older adults are more likely to live in multigenerational or skipped generation households. Indigenous older adults are more likely to live in multigenerational households. Most of the older adult population has lower levels of education (less than high school/no schooling), but those living alone or only with older adults are more likely to have a college education or higher. Households composed of older adults are more prevalent among the third and fourth quartiles of household income than households without older adults. Per capita household income is even higher for older adults living independently or with only other older adults. Multigenerational and skipped generation households are more prevalent in more impoverished regions in Brazil (i.e., North and Northeast), whereas independent living is more prevalent in more developed areas. Older adults generally have a high prevalence of hypertension and diabetes, and older adults living alone have worse health than those in other living arrangements.

Table 2 shows the prevalence of COVID-19 symptoms, testing, and positive results across individuals’ characteristics. We observe significant variation across living arrangements across all our COVID-19 related outcomes. Regarding subjective assessments of COVID-19 exposure, older adults living with other people were less likely to report having COVID-19 symptoms than older adults living alone. Regarding overall testing, older adults living in multigenerational (PR = 0.82, 95% CI 0.73 3, 0.92) or skipped generation households (PR = 0.73, 95% CI 0.60,0.89) were less likely to test than older adults living alone.

When we consider having positive COVID test results, however, older adults living with children and/or adults were more likely to test positive during the pandemic. Those living in multigenerational households were more likely to test positive (the prevalence was 53% higher than those living alone, all else equal). Compared to those living alone, older adults living in a skipped generation household had a 61% higher prevalence of positive tests, and those living with adults had a 27% higher prevalence. However, older adults living with other older adults did not differ from older adults living alone in testing positive for COVID-19.

Table 3 examines how differential self-reporting of symptoms across living arrangements may influence the testing prevalence and positive cases. The results show that self-reporting symptoms were associated with a higher prevalence of testing for COVID-19. In addition, interaction terms indicate that having symptoms modifies the association between living arrangements and testing. Figure 2 shows these effects. Among older adults with symptoms, we do not observe statistically significant differences in testing across living arrangements. However, among older adults without symptoms, those living alone had a higher probability of testing than those living in multigenerational or skipped-generation households.

Finally, regarding receiving a positive test result, those with symptoms were more likely to be positive. Compared to older adults living alone, older adults living in multigenerational or skipped households were particularly more likely to receive positive COVID-19 test results. However, self-reported symptoms were not a statistically significant moderator in the association between living arrangements and positive test results.

Last, we examined whether results based on the cumulative measures of symptoms, testing, and positive results across all months yielded similar conclusions (see Tables 4 and 5). The findings are broadly in agreement with the results shown in Tables 2 and 3. Table 4 shows that older adults living alone were more likely to report having symptoms during July–November of 2020 and more likely to test than older adults living in multigenerational or skipped generation households. However, older adults living with other generations, being children and/or adults, were more likely to receive a positive result.

**Table 4:** Adjusted odds ratios and 95% confidence intervals (CI) for factors associated with the cumulative reporting of COVID-19 symptoms, testing and receiving a positive result, older adults, Brazil

	Symptoms		Testing		Positive	
	OR	95% CI	OR	95% CI	OR	95% CI
*Fixed*						
Living arrangement (versus older adult alone)						
Older adults only	0.737***	(0.69, 0.79)	0.958	(0.88, 1.04)	1.073	(0.90, 1.28)
Multigenerational	0.705***	(0.65, 0.77)	0.828***	(0.75, 0.91)	1.275*	(1.05, 1.55)
Skipped generation	0.774***	(0.67, 0.89)	0.789**	(0.67, 0.94)	1.459*	(1.08, 1.96)
Adults and older adults	0.724***	(0.68, 0.78)	0.937	(0.86, 1.02)	1.196*	(1.01, 1.42)
Age (in years)	0.999	(1.00, 1.00)	0.991***	(0.99, 0.99)	0.986***	(0.98, 0.99)
Female (versus male)	1.033	(0.99, 1.08)	0.946*	(0.90, 0.99)	0.938	(0.86, 1.03)
Race (versus White)						
Black	1.103*	(1.02, 1.20)	0.990	(0.90, 1.08)	0.973	(0.81, 1.17)
Brown	1.081**	(1.03, 1.13)	0.978	(0.93, 1.03)	1.067	(0.96, 1.18)
Indigenous	1.609**	(1.15, 2.24)	1.427*	(1.00, 2.03)	1.502	(0.82, 2.75)
Other	1.069	(0.85, 1.35)	0.941	(0.73, 1.21)	0.655	(0.36, 1.20)
Education (versus no schooling)						
Less than HS	0.977	(0.92, 1.04)	1.049	(0.97, 1.13)	1.088	(0.94, 1.26)
HS complete	0.905*	(0.84, 0.98)	1.285***	(1.18, 1.41)	1.417***	(1.19, 1.69)
College or more	0.839***	(0.76, 0.92)	1.705***	(1.55, 1.88)	1.456***	(1.20, 1.77)
Per capita household quartile (versus 1st)						
2	0.970	(0.91, 1.03)	0.952	(0.88, 1.03)	1.031	(0.88, 1.20)
3	0.958	(0.90, 1.02)	1.130**	(1.05, 1.22)	1.257**	(1.08, 1.47)
4	0.894**	(0.83, 0.96)	1.426***	(1.32, 1.54)	1.564***	(1.33, 1.84)
Region (versus North)						
Northeast	0.850***	(0.78, 0.92)	0.871**	(0.80, 0.95)	0.623***	(0.54, 0.72)
Southeast	0.746***	(0.69, 0.81)	0.590***	(0.54, 0.64)	0.334***	(0.29, 0.39)
South	0.879**	(0.81, 0.96)	0.525***	(0.48, 0.58)	0.259***	(0.22, 0.31)
Center West	0.950	(0.86, 1.04)	0.960	(0.87, 1.06)	0.693***	(0.59, 0.82)
Rural (versus urban)	0.952	(0.90, 1.00)	0.589***	(0.55, 0.63)	0.609***	(0.53, 0.70)
Health conditions (versus none)						
Diabetes	1.217***	(1.16, 1.28)	1.125***	(1.06, 1.19)	1.281***	(1.15, 1.43)
Hypertension	1.210***	(1.16, 1.26)	1.043	(0.99, 1.09)	1.175***	(1.07, 1.29)
Heart disease	1.437***	(1.35, 1.53)	1.437***	(1.06, 1.23)	1.097	(0.95, 1.27)
Asthma	2.099***	(1.95, 2.26)	1.355***	(1.24, 1.48)	1.231*	(1.03, 1.48)
Cancer	1.300***	(1.17, 1.44)	1.173**	(1.05, 1.32)	1.142	(0.91, 1.44)
Depression	1.767***	(1.63, 1.91)	1.157**	(1.05, 1.28)	1.229*	(1.01, 1.49)
Proxy respondent (versus no)	0.665***	(0.64, 0.70)	0.928**	(0.88, 0.98)	0.964	(0.87, 1.06)
Constant	0.37	(0.30, 0.47)	0.05	(0.31, 0.52)	0.10	(0.06, 0.17)

Source: Authors’ calculations using data from the COVID-19 PNAD, cumulative July–November. Robust standard errors

Note: * *p* < 0.05, ** *p* < 0.01, *** *p* < 0.001, 95% CI: 95% confidence interval

**Table 5:** Adjusted Odds Ratios and 95% confidence intervals (CI) for factors associated with the cumulative reporting of COVID-19 testing and receiving a positive result, Brazil (*n* = 63,268)

	Testing (Model 1)		Testing (Model 2)		Positive (Model 1)		Positive (Model 2)	
	PR	95% CI	PR	95% CI	PR	95% CI	PR	95% CI
Living arrangement (versus older adult alone)								
Older adults only	1.023	(0.94, 1.11)	0.919	(0.83, 1.01)	1.221*	(1.02, 1.47)	0.920	(0.71, 1.19)
Multigenerational	0.882*	(0.80, 0.98)	0.764***	(0.68, 0.86)	1.455***	(1.19, 1.78)	1.241	(0.95, 1.63)
Skipped generation	0.828*	(0.70, 0.98)	0.702**	(0.57, 0.87)	1.629**	(1.20, 2.20)	1.209	(0.77, 1.91)
Adults and older adults	0.999	(0.92, 1.09)	0.897*	(0.81, 0.99)	1.358***	(1.14, 1.62)	1.089	(0.85, 1.40)
Symptoms	2.832***	(2.69, 2.98)	2.184***	(1.92, 2.48)	5.852***	(5.32, 6.44)	4.167***	(3.14, 5.53)
Interactions								
Symptoms*older adults only			1.328***	(1.13, 1.56)			1.632**	(1.16, 2.29)
Symptoms*multigenerational			1.515***	(1.26, 1.83)			1.266	(0.89, 1.80)
Symptoms*skipped generation			1.560*	(1.09, 2.22)			1.662	(0.91, 3.03)
Symptoms*adults and older adults			1.327***	(1.14, 1.54)			1.447*	(1.06, 1.98)
Age (in years)	0.991***	(0.99, 0.99)	0.991***	(0.99, 0.99)	0.987***	(0.98, 0.99)	0.987***	(0.98, 0.99)
Female (versus male)	0.937**	(0.89, 0.98)	0.936**	(0.89, 0.98)	0.921	(0.84, 1.01)	0.921	(0.84, 1.01)
Race (versus White)								
Black	0.974	(0.89, 1.07)	0.975	(0.89, 1.07)	0.942	(0.78, 1.13)	0.942	(0.78, 1.13)
Brown	0.965	(0.91, 1.02)	0.964	(0.91, 1.02)	1.042	(0.94, 1.16)	1.040	(0.94, 1.16)
Indigenous	1.305	(0.91, 1.88)	1.294	(0.90, 1.87)	1.231	(0.66, 2.28)	1.225	(0.66, 2.27)
Other	0.932	(0.72, 1.20)	0.932	(0.72, 1.20)	0.651	(0.36, 1.18)	0.653	(0.36, 1.18)
Education (versus no schooling)								
Less than HS	1.057	(0.98, 1.14)	1.057	(0.98, 1.14)	1.103	(0.95, 1.28)	1.104	(0.95, 1.28)
HS complete	1.319***	(1.21, 1.44)	1.319***	(1.20, 1.44)	1.478***	(1.24, 1.76)	1.478***	(1.24, 1.76)
College or more	1.794***	(1.62, 1.98)	1.792***	(1.62, 1.98)	1.581***	(1.29, 1.93)	1.584***	(1.30, 1.94)
Per capita household quartile (versus 1st)								
2	0.959	(0.89, 1.04)	0.956	(0.88, 1.03)	1.048	(0.89, 1.23)	1.046	(0.89, 1.23)
3	1.149***	(1.06, 1.24)	1.146***	(1.06, 1.24)	1.302**	(1.11, 1.53)	1.300**	(1.11, 1.52)
4	1.476***	(1.36, 1.60)	1.473***	(1.36, 1.60)	1.667***	(1.41, 1.97)	1.669***	(1.41, 1.97)
Region (versus North)								
Northeast	0.890**	(0.82, 0.97)	0.889**	(0.82, 0.97)	0.640***	(0.55, 0.74)	0.640***	(0.55, 0.74)
Southeast	0.612***	(0.56, 0.67)	0.612***	(0.56, 0.67)	0.355***	(0.30, 0.41)	0.355***	(0.30, 0.41)
South	0.524***	(0.48, 0.58)	0.524***	(0.48, 0.58)	0.255***	(0.21, 0.31)	0.254***	(0.21, 0.31)
Center West	0.966	(0.88, 1.07)	0.967	(0.88, 1.07)	0.690***	(0.58, 0.82)	0.690***	(0.58, 0.82)
Rural (versus urban)	0.587***	(0.55, 0.63)	0.587***	(0.55, 0.63)	0.612***	(0.54, 0.70)	0.612***	(0.53, 0.70)
Health conditions (versus none)								
Diabetes	1.087**	(1.03, 1.15)	1.087**	(1.03, 1.15)	1.198**	(1.07, 1.34)	1.198**	(1.07, 1.34)
Hypertension	1.010	(0.96, 1.06)	1.010	(0.96, 1.06)	1.103*	(1.00, 1.22)	1.103*	(1.00, 1.22)
Heart disease	1.060	(0.98, 1.14)	1.061	(0.98, 1.14)	1.060	(0.82, 1.11)	0.954	(0.82, 1.11)
Asthma	1.167**	(1.06, 1.28)	1.172***	(1.07, 1.29)	0.937	(0.78, 1.13)	0.937	(0.78, 1.13)
Cancer	1.119	(1.00, 1.26)	1.122	(1.00, 1.26)	1.044	(0.83, 1.32)	1.047	(0.83, 1.32)
Depression	1.022	(0.92, 1.13)	1.027	(0.93, 1.14)	0.979	(0.80, 1.20)	0.980	(0.80, 1.20)
Proxy respondent (versus no)	0.997	(0.95, 1.05)	1.000	(0.95, 1.05)	1.119*	(1.01, 1.24)	1.120*	(1.01, 1.24)
Constant	0.280	(0.21, 0.37)	0.311	(0.24, 0.41)	0.046	(0.03, 0.08)	0.057	(0.03, 0.10)

Source: Authors’ calculations using data from the COVID-19 PNAD, cumulative July–November. Robust standard errors

Note: * *p* < 0.05, ** *p* < 0.01, *** *p* < 0.001, 95% CI: 95% confidence interval

Results presented in Table 5 confirm that having symptoms was associated with higher odds of testing (as shown in Table 3). Although older adults living alone were more likely to test than those in multigenerational or skipped households, results based on the cumulate measures show statistically significant interactions between living arrangements and symptoms. Findings indicate higher increases in testing among older adults with symptoms in living arrangements with others, as highlighted in Fig. 2. In sum, older adults living alone without symptoms were more likely to test than their counterparts living with adults or children. However, among those with symptoms, older adults living with other older adults or with adults were more likely to test than those living alone. These results differed slightly from those in Table 3 and highlighted in Fig. 2, as examining cumulative measures reveals only older adults living with other older adults or adults were more likely to test than those living alone.

Finally, having a positive result was more common in all living arrangements than living alone and higher among those with symptoms. Contrasts indicate that among those with symptoms, older adults living with others were more likely to test positive at some point during the selected months than those living alone.

## Discussion

Brazil has been devastated by the COVID-19 pandemic. Social distancing measures, a crucial means to mitigate the spread of the virus prior to the wide availability of vaccines, are potentially less effective in Brazil as intergenerational coresidence and interdependence of support across generations are commonplace. Against this background, our study aimed to identify which living arrangements present the most health risks to older adults in Brazil.

### Living arrangements, COVID-19 symptoms, and testing

Our findings show that older adults living alone were more likely to report symptoms of COVID-19 than older adults with any other living arrangements. These associations were evident even after controlling for older adults’ self-report of chronic health conditions that correlate with susceptibility to COVID-19. If COVID-19 symptoms are an indicator of older adults’ subjective health, our findings align with research in other developing countries where multigenerational living arrangements prevail, which shows that solo-dwelling presents a health risk for older adults in these contexts [34–36]. However, the disrupted access to formal and informal support services in Brazil during the pandemic complicates this view. Older adults living alone may have had heightened overall concerns about potential exposure to the virus and awareness of the importance of maintaining their health because they live independently, and these concerns might increase their likelihood of reporting COVID-19 symptoms. This heightened concern and self-reliance may also play a role in the higher likelihood of testing among solo-dwelling older adults without symptoms relative to those who live with others.

Self-reported symptoms were positively associated with COVID-19 testing overall. However, having symptoms had differential effects on testing across living arrangements. While those without symptoms were more likely to test if they lived alone—albeit at lower rates than any group with symptoms—those with symptoms were more likely to test if they lived with other adults. As other studies have observed [37], coresidence with family members may facilitate various types of support, including financial, informational, and instrumental support, and such support may allow older adults with symptoms to test. Another possible factor is that older adults living in larger households may be more likely to test for COVID-19 once they have symptoms because of concerns about exposures within the household. In fact, in results not shown, the prevalence of testing among older adults was 99% higher when another family member reported symptoms in the household. Although it is beyond the scope of our study to determine the underlying mechanisms that explain testing among older adults in larger households relative to those living alone, our results suggest that coresidence with other family members facilitates access to potentially preventive health service – COVID-19 testing – among those with symptoms.

### Intergenerational Coresidence and risks of COVID-19 positivity

Living in multigenerational and skipped generation households, or households with other adults, was associated with the highest likelihood of testing positive for COVID-19. Consistent with prior research on living arrangements and older adults’ risks of other infectious diseases [34], these findings suggest that coresidence with younger family members can present an elevated risk of exposure to COVID-19 as measured by positive testing and represent a health risk for older adults. Taken together, our findings confirm expectations by other scholars [16] that residing with younger generations presents a health risk in the context of the ongoing pandemic.

Whereas more developed countries have reported changes in the living arrangements of older adults [65], the living arrangements in Brazil were remarkably stable over the observed months, with 96% of the older adults being in the same living arrangement in November as they had in July. Therefore, older adults in Brazil, particularly those who lived in larger households, had few opportunities to protect against the heightened risks related to the presence of others in the home [66]. These risks are higher for those sharing the household with adults employed in essential services, and thus were working outside the household throughout the pandemic. Risks of having a positive result are also higher for older adults living with adult children, and those only living with their grandchildren, who may be asymptomatic but transmit to the older adult. These households with more than one generation facilitate the transmission of the disease as the ability to social distance and isolate becomes more challenging as more people share the home. Multigenerational households are often crowded, having more than 3.5 persons per household [67]. Furthermore, these extended multigenerational living arrangements are common among the poor, Black, and Brown populations, thus compounding inequalities in health vulnerabilities.

### Limitations

This study has some limitations. First, all the data are self-reported. COVID-19 symptoms may be associated with health conditions other than COVID-19. Although our models control for disease risk through self-reported chronic conditions, respondents might have health conditions unknown to them that can increase susceptibility to COVID-19. Additionally, positive COVID-19 results depend on testing, which varies across social groups and geographic areas. Even though testing availability increased in Brazil, it has not been sufficient to identify all cases. Thus, our data may still underestimate the relationship between living arrangements and COVID-19 positive results due to many undetected cases for lack of testing. Moreover, while reporting by a single adult member of the household is a standard procedure for household surveys in Brazil, such reporting may not be entirely objective or reliable. Second, although our analyses cover 5 months in 2020, the pandemic worsened in Brazil after November and continues to take many lives. At the time of writing, younger people are more likely to contract the virus and experience severe health outcomes than older people [68]. This may have changed some of the dynamics we examined here. Third, the PNAD-C does not provide a unique individual identifier, so we cannot track individuals across the waves. Finally, our analyses focus on structural dimensions of social support in the form of living arrangements, and we cannot examine other dimensions of support, including intergenerational transfers from coresident and non-resident family members. Although we find evidence of differential health risks of COVID-19, as indicated by positive test results, for older adults living with younger cohorts relative to those living alone, we cannot assess the underlying direct mechanisms. For instance, solo-dwelling older adults may still receive support transfers from non-resident family members, which may facilitate their access to testing and confirm or contradict their self-reported symptoms. Future research should incorporate direct assessment of support transfers for older adults’ COVID-19 risks linked to their living arrangements.

## Conclusions

Our study has several major strengths, namely the use of a nationally representative sample of older adults that we were able to assess longitudinally during a significant period of the COVID-19 pandemic in Brazil. By examining the role of diverse living arrangements for older adults’ vulnerability to COVID-19, and their access to COVID-19 specific health care by means of testing, we contribute to the empirical evidence on COVID-19 vulnerability in Brazil. Previous studies have addressed how older adults with symptoms have sought care [61], but the impact of living arrangements and family composition on COVID-19 outcomes have been understudied more generally [16].

As the federal government has neglected to address the challenges the population faces, civil society groups and organizations have organized to support people who need to self-isolate [69]. However, the government should support older adults and their families. For older adults who live alone, policies that increase safety when shopping for groceries, such as mask requirements and specific times for older adults, well-being checks, and programs to combat loneliness, could help. For those who live with others, particularly in crowded situations, policies aimed at providing places for safe isolation and financial resources for family members who provide care to stay home have the potential to help contain the spread of the disease. Alternative ways to help older adults manage chronic conditions, such as telemedicine, could also help maintain their health. Ultimately, increased funding for the Unified Health System is needed to treat COVID-19 cases, as 80% of older adults depend on the public system for health care [70]. The results of this study could aid in targeting the resulting increased services, as it identifies areas of need among this vulnerable population according to living arrangements.

## Data Availability

All the data can be obtained from the Brazilian Census Bureau, Instituto Brasileiro de Geografia e Estatistica (ibge.gov.br), website (https://covid19.ibge.gov.br/pnad-covid/).

## Abbreviations

CI: Confidence Interval
COVID-19: Coronavirus Disease of 2019
PNAD: Pesquisa Nacional por Amostra de Domicílios [National Household Survey]
PNAD-C: Pesquisa Nacional por Amostra de Domicílios Continua [Continuous National Household Survey]
PR: Prevalence Ratio
US: United States
